# Upregulation of Thymidylate Synthase Induces Pemetrexed Resistance in Malignant Pleural Mesothelioma

**DOI:** 10.3389/fphar.2021.718675

**Published:** 2021-09-27

**Authors:** Yuzo Sato, Masaru Tomita, Tomoyoshi Soga, Atsushi Ochiai, Hideki Makinoshima

**Affiliations:** ^1^ Tsuruoka Metabolomics Laboratory, National Cancer Center, Tsuruoka, Japan; ^2^ Shonai Regional Industry Promotion Center, Tsuruoka, Japan; ^3^ Systems Biology Program, Graduate School of Media and Governance, Keio University, Fujisawa, Japan; ^4^ Institute for Advanced Biosciences, Keio University, Tsuruoka, Japan; ^5^ Exploratory Oncology Research and Clinical Trial Center, National Cancer Center, Kashiwa, Japan; ^6^ Division of Translational Information, Exploratory Oncology Research and Clinical Trial Center, National Cancer Center, Kashiwa, Japan

**Keywords:** drug-resistance, H3K27ac, mesothelioma, thymidylate synthase, tumor metabolism

## Abstract

Malignant pleural mesothelioma (MPM) is an invasive malignancy that develops in the pleural cavity, and antifolates are used as chemotherapeutics for treating. The majority of antifolates, including pemetrexed (PMX), inhibit enzymes involved in purine and pyrimidine synthesis. MPM patients frequently develop drug resistance in clinical practice, however the associated drug-resistance mechanism is not well understood. This study was aimed to elucidate the mechanism underlying resistance to PMX in MPM cell lines. We found that among the differentially expressed genes associated with drug resistance (determined by RNA sequencing), TYMS expression was higher in the established resistant cell lines than in the parental cell lines. Knocking down *TYMS* expression significantly reduced drug resistance in the resistant cell lines. Conversely, *TYMS* overexpression significantly increased drug resistance in the parental cells. Metabolomics analysis revealed that the levels of dTMP were higher in the resistant cell lines than in the parental cell lines; however, resistant cells showed no changes in dTTP levels after PMX treatment. We found that the nucleic acid-biosynthetic pathway is important for predicting the efficacy of PMX in MPM cells. The results of chromatin immunoprecipitation-quantitative polymerase chain reaction (ChIP-qPCR) assays suggested that H3K27 acetylation in the 5′-UTR of *TYMS* may promote its expression in drug-resistant cells. Our findings indicate that the intracellular levels of dTMP are potential biomarkers for the effective treatment of patients with MPM and suggest the importance of regulatory mechanisms of TYMS expression in the disease.

## Introduction

Malignant pleural mesothelioma (MPM) is a locally invasive and fatal malignancy associated with asbestos exposure (Liu et al., 2017; Yap et al., 2017). MPM develops in the pleural cavity, is highly resistant to several therapeutics, and is associated with a poor patient prognosis (Creaney and Robinson, 2017; Liu et al., 2017; Scherpereel et al., 2018). Combination treatment with pemetrexed (PMX, also known as Alimta and LY231514) and cisplatin has been the first-line chemotherapy regimen for more than a decade (Vogelzang et al., 2003; Scagliotti et al., 2008; Liu et al., 2017). PMX is an antifolate that simultaneously inhibits the synthesis of both purines and pyrimidines (Shih et al., 1997). PMX and its polyglutamated derivatives inhibit thymidylate synthase (*TYMS*), dihydrofolate reductase (*DHFR*), and glycinamide ribonucleotide transformylase (*GART*), all of which are involved in the *de novo* biosynthesis of thymidine and purine nucleotides (Shih et al., 1997; Yap et al., 2017). Antimetabolite agents, including PMX, induce an imbalance in the cellular nucleotide pool and inhibit nucleic acid biosynthesis, resulting in arrested tumor cell proliferation and tumor cell death (Zhao and Goldman, 2003; Yap et al., 2017). Clinically, treatment with a combination of PMX and cisplatin, along with vitamin supplements, resulted in a superior survival period, time to progression, and response rates compared to treatment with cisplatin alone in patients with MPM (Vogelzang et al., 2003; Scagliotti et al., 2008). However, the median survival period was approximately 12 months, and the response rate was 41.3% after treatment with PMX and cisplatin (Vogelzang et al., 2003). This indicates that approximately 60% of patients with MPM are resistant to PMX and cisplatin (Zhu et al., 2016).

Genes related to antifolate resistance include *DHFR*, *TYMS*, ATP-binding cassette subfamily C (*ABCC*), and reduced folate carrier (*RFC*) (Wang et al., 2003; Zhao and Goldman, 2003; Assaraf, 2007). Many of these genes are upregulated by long-term drug exposure, resulting in drug resistance. TYMS, a metabolic enzyme, catalyzes the methylation of deoxyuridine monophosphate (dUMP) to deoxythymidine monophosphate (dTMP) in thymidylate biosynthesis (Danenberg, 1977). This enzyme, has the affinity for PMX, is understood as a primary target for PMX, and is recognized as an important target gene in chemotherapy for several cancers (Liu et al., 2019; Varghese et al., 2019; Song et al., 2021). Previous reports have indicated the potential involvement of genes such as MYC proto-oncogene, bHLH transcription factor (*MYC*), forkhead box M1 (*FOXM1*), and E2F transcription factor 1 (*E2F1*) in regulating TYMS expression; in some cancers, TYMS expression was upregulated by *FOXM1* and *MYC* and downregulated by *E2F1* (Lam et al., 2014; Liu et al., 2019; Varghese et al., 2019). However, the details of the underlying mechanisms remain unknown. TYMS overexpression was previously associated with PMX resistance (Sigmond et al., 2003; Takezawa et al., 2011; Zhang et al., 2011). In contrast, another reports suggested that TYMS overexpression was not linked to clinical outcomes (Kitazono-Saitoh et al., 2012). Taken together, the mechanism of PMX resistance is not understood in detail.

We previously reported that the levels of glycine and inosine monophosphate are potential biomarkers of the efficacy of PMX chemotherapy (Sato et al., 2018). Moreover, as previous study revealed that extracellular dTTP or thymidine rescues the antiproliferative effects of PMX (Yang et al., 2013; Sato et al., 2018). Therefore, we hypothesize that intracellular metabolites might be affected by acquisition of drug resistance.

In this study, we aimed to elucidate the role of TYMS in the development of drug resistance using MPM cell lines. We evaluated the expression levels of TYMS and related genes in the MPM cells, and we assessed the effect of PMX on intracellular metabolite concentrations in MPM cells.

## Materials and Methods

### Materials

RPMI-1640 (R8758), Dulbecco’s modified Eagle’s medium (DMEM; D5796), and phosphate-buffered saline (PBS) were purchased from Sigma-Aldrich (St. Louis, MO, United States). Fetal bovine serum (FBS) was purchased from Biowest (Nuaille, France). Dimethyl sulfoxide was purchased from FUJIFILM Wako Pure Chemicals Corporation (Osaka, Japan). PMX and 5-fluorouracil (5-FU) were purchased from Selleck Chemicals (Houston, TX, United States). Puromycin, blasticidin, and geneticin were purchased from Thermo Fisher Scientific (Waltham, MA, United States). Cell Counting Kit-8 was purchased from Dojindo Laboratories (Kumamoto, Japan). Mini-PROTEAN TGX Precast Gels, the Trans-Blot Turbo Transfer System, and Trans-Blot Turbo Transfer Packs were purchased from Bio-Rad Laboratories, Inc. (Hercules, CA, United States). For western blotting, primary antibodies against DHFR (#ab124814), GART (#ab169550), TYMS (#ab108995), FOXM1 (#ab207298), E2F1 (#ab179445), and β-tubulin (TUBB, #ab179513) were purchased from Abcam (Cambridge, United Kingdom), and those against c-MYC (#5605), acetyl-histone H3 (Lys27) (H3K27ac, #8173), and tri-methyl-histone H3 (Lys4) (H3K4me3, #9727) were purchased from Cell Signaling Technology (CST; Danvers, MA, United States). Horseradish peroxidase (HRP)-linked secondary, whole-antibody donkey anti-rabbit IgG (#NA934) was purchased from Cytiva (Pittsburgh, PA, United States) and used for western blotting. SYBR Premix Ex Taq and specific primers (TYMS, #HA147396; DHFR, #HA147396; GART, #HA283720; glyceraldehyde 3-phosphate dehydrogenase [GAPDH], #HA031578) were purchased from TaKaRa Bio (Shiga, Japan). Lipofectamine RNAiMAX Transfection Reagent was purchased from Invitrogen (Carlsbad, CA, United States). FuGENE HD was purchased from Promega (Madison, WI, United States).

### Cell Lines and Cell Culture

MPM is divided into three main histological subtypes: epithelioid, sarcomatoid, and biphasic. The epithelioid and sarcomatoid subtypes are characterized by cuboid and fibroblastoid cells, respectively, whereas the biphasic subtype contains a mixture of both cell types. A commercially available MSTO-211H (biphasic) cell line was purchased from the American Type Culture Collection (Manassas, VA, United States). The TCC-MESO-2 (epithelial) cell line was established from Japanese patients with MPM, and its biological characteristics have been previously reported (Yanagihara et al., 2010). The Platinum-A Retroviral Packaging Cell Line (Plat-A) was purchased from Cell Biolabs, Inc. (San Diego, CA, United States). MPM cell lines were cultured in RPMI-1640 medium supplemented with 10% FBS. Plat-A was cultured in DMEM supplemented with 10% FBS, puromycin (1 μg/ml) and blasticidin (10 μg/ml).

### Cell Survival and Cell-Proliferation Assays

MPM cells were seeded in RPMI-1640 medium containing different concentrations of PMX (1 nM–100 μM) or 5-FU (100 pM–10 μM) in 96-well cell culture plates. After incubation for 72 h, cell viability was analyzed by performing water-soluble tetrazolium salt-8 (WST-8) assays using the Cell Counting Kit-8. The PMX or 5-FU concentrations were plotted against the percentages of surviving cells for all MPM cell lines, and the respective half-maximal inhibitory concentration (IC_50_) values were calculated using GraphPad Prism 8 software (GraphPad Software, Inc., La Jolla, CA, United States).

### Resistance Induction

MPM cells were plated at a density of 8 × 10^5^ cells per dish. After 24 h, the medium was replaced with containing PMX at the concentration of IC_50_ (MSTO-211H = 47.4 nM, TCC-MESO-2 = 94.3 nM), as described previously (Sato et al., 2018). The medium was changed every 2 days, the concentration of PMX was increased by 10% at each medium change. When the cells reached 80% confluence, the PMX concentration was doubled. However, the medium was replaced with complete medium when the confluence was less than 50%. These steps were repeated until the concentration reached 10 μM. The established resistant cell lines, named MSTO-211H_R and TCC-MESO-2_R, were cultured in the presence of 10 μM PMX. The IC_50_ values were determined for both resistant cell lines, as described above.

### Reverse Transcription-Quantitative Polymerase Chain Reaction Analysis

MPM cells were washed with PBS, and total RNA was isolated using the TRIzol Reagent (Invitrogen) following the manufacturer’s protocol. Complementary DNA (cDNA) was synthesized using RNA samples (1 μg) and the SuperScript VILO cDNA Synthesis Kit (Invitrogen). The mRNA-expression levels of each target gene were determined using specific primers (TaKaRa Bio), SYBR Premix Ex Taq (TaKaRa Bio), and a QuantStudio 3 Real-Time PCR System (Thermo Fisher Scientific). All expression data were normalized to GAPDH levels using the comparative CT method, according to the manufacturer’s protocol.

### Western Blotting

Cells were lysed with Cell Lysis Buffer (CST) on ice for 2 min and centrifuged at 15,000 × *g* for 10 min. The supernatant protein contents were measured by performing bicinchoninic assays using the Pierce BCA Protein Assay Kit (Thermo Fisher Scientific). Equivalent amounts of protein samples were separated by 4–20% sodium dodecyl sulfate-polyacrylamide gel electrophoresis, transferred to polyvinylidene fluoride membranes, and incubated at 4°C overnight with primary antibodies (1:1,000 dilution). An HRP-linked whole-antibody donkey anti-Rabbit IgG (1:10,000; Cytiva) was used as the secondary antibody. Signals were detected using the ECL Prime Western Blotting Detection Reagent (Cytiva) and FUSION FX imager (Vilber, Collégien, France). The band intensities were quantified using Fusion Capt Advance FX7 software (Vilber).

### RNA Sequencing

MPM cells were seeded at 1 × 10^6^ cells per dish. After 24 h, MPM cells were treated with PMX (1 μM) or PBS for 6 h. Extracted total RNA was sequenced by Macrogen (Tokyo, Japan). Sequencing was performed using the TruSeq Stranded mRNA LT Sample Prep Kit and the NovaSeq 6000 System (Illumina, San Diego, CA, United States).

### Small-Interfering RNA Transfection

siRNA transfections were performed using Silencer™ Select Pre-Designed siRNA against TYMS (siTYMS; cat#4392421, ID#s14539) and Silencer™ Select Negative Control siRNA (catalog #4390844), as a negative control (NC), purchased from Thermo Fisher Scientific. Six-well plates were seeded at 25,000 cells/well and transfections were performed using the Lipofectamine RNAiMAX reagent following the manufacturer’s instructions. MSTO-211H_R and TCC-MESO-2_R cells were transfected with 10 nM or 1 nM siTYMS, respectively. After 48 h incubation, the cells were harvested for western blotting and cell-survival assays.

### Overexpression Assay

For *TYMS* overexpression, MPM cell lines were transfected using the Plat-A retroviral packaging cell line. Initially, FuGENE HD was used to transfect the packaging cell line with one of two plasmids (Unitec, Chiba, Japan), i.e., a control plasmid (pMXs-Neo-Vector) or a plasmid encoding the target gene (pMXs-Neo-TYMS), to produce retroviruses. Twenty-four hours after transfection, the MPM cells were transduced with either retrovirus. After 48 h, the cells were cultured in RPMI-1640 supplemented with 10% FBS and geneticin (750 μg/ml).

### Chromatin Immunoprecipitation

For ChIP experiments, chromatin was extracted from parental and resistant cells using the SimpleChIP Enzymatic Chromatin IP Kit (#9003; CST). After extraction, ChIP was performed with 5 µg of anti-histone H3 (#4620; CST), anti-normal rabbit IgG (#2792; CST), anti-H3K4me3 (CST), and anti-H3K27ac (CST) antibodies against 5 µg of chromatin, according to the manufacturer’s protocol. The purified DNA fragments were quantitated by qPCR, performed on a QuantStudio 3 Real-Time PCR System using the SimpleChIP Universal qPCR Master Mix (#88989; CST), included primers in the SimpleChIP Enzymatic Chromatin IP Kit and specific custom-made primer (FW; CCT​GGC​GGT​TTT​TAA​TCA​AG, R; CAC​AGT​TCC​CAC​GTT​TTC​CT) (Varghese et al., 2019), according to the manufacturer’s protocol. The input DNA was diluted to 2%, and the qPCR data were normalized to input DNA and analyzed using the following formula (%):

$$\mathrm{Percent}\;\mathrm{input=2\%}\left(\mathrm{DNA}\;\mathrm{input}\;\mathrm{dilution}\;\mathrm{factor}\right)\times 2^{\wedge }\left(\mathrm{Ct}\left[\mathrm{input}\;\mathrm{DNA}\right]\mathrm{-Ct}\left[\mathrm{ChIP}\right]\right)$$

where Ct = the cycle threshold of each target gene

### Metabolite Measurements

MPM cells were treated with PBS or PMX for 6 h. Metabolic extracts were prepared from 1 × 10^6^ MPM cells with methanol containing an internal standard solution and analyzed using capillary electrophoresis-time-of-flight mass spectrometry (CE-TOFMS) and capillary electrophoresis-tandem mass spectroscopy (CE-MS/MS). d-camphor-10-sulfonic acid (FUJIFILM Wako Pure Chemicals Corporation) was used as the internal standard. Cells were washed twice in 5% mannitol solution and then treated with 1 ml of methanol containing 25 µM internal standard solution. Metabolite extract (400 μl) was transferred into a new microfuge tube, 400 μl chloroform and 200 μl Milli-Q water were added, and the resulting solution was mixed well and centrifuged at 10,000 × g for 3 min at 4°C. The upper aqueous layer was centrifugally filtered through a 5 kDa-cutoff filtration column (Ultrafree MC-PLHCC 250/pk for Metabolome Analysis; #UFC3LCCNB-HMT, Human Metabolome Technologies, Inc., Japan) to eliminate proteins. The filtrate was centrifugally concentrated and resuspended in 50 µl Milli-Q water for CE-MS analysis. Cationic compounds were analyzed CE-TOFMS in positive-ion mode, and anionic compounds were analyzed by CE-MS/MS in positive- and negative-ion modes, according to the methods developed by Soga and Heiger (2000), Soga et al. (2002), Soga et al. (2003). To evaluate peak information, including the mass: charge ratio, migration time, and peak area, the peaks detected by CE-TOFMS and CE-MS/MS were extracted using automatic integration software (MasterHands, Keio University, Tsuruoka, Japan and MassHunter Quantitative Analysis B.06.00, Agilent Technologies, Santa Clara, CA, United States, respectively). Metabolite concentrations were calculated by normalizing the peak area of each metabolite to that of the internal standard, using standard curves obtained by three-point calibrations.

### Statistical Analyses

The results are presented as the mean ± standard deviation, unless indicated otherwise. Statistical analyses were performed using Welch’s t-test, and *p*-values <0.05, <0.01, and <0.001 were considered to reflect significant differences.

### Availability of Data

All RNA-seq data have been uploaded in the DNA Data Bank of Japan (DDBJ, Mishima, Shizuoka, Japan). DDBJ sequence read archive (DRA) accession number: DRA012402, DRA012403, DRA012404, DRA012405, DRA012406.

## Results

### IC_50_ Measurement in Pemetrexed-Treated Parental and Pemetrexed-Resistant Cell Lines

**FIGURE 1:**
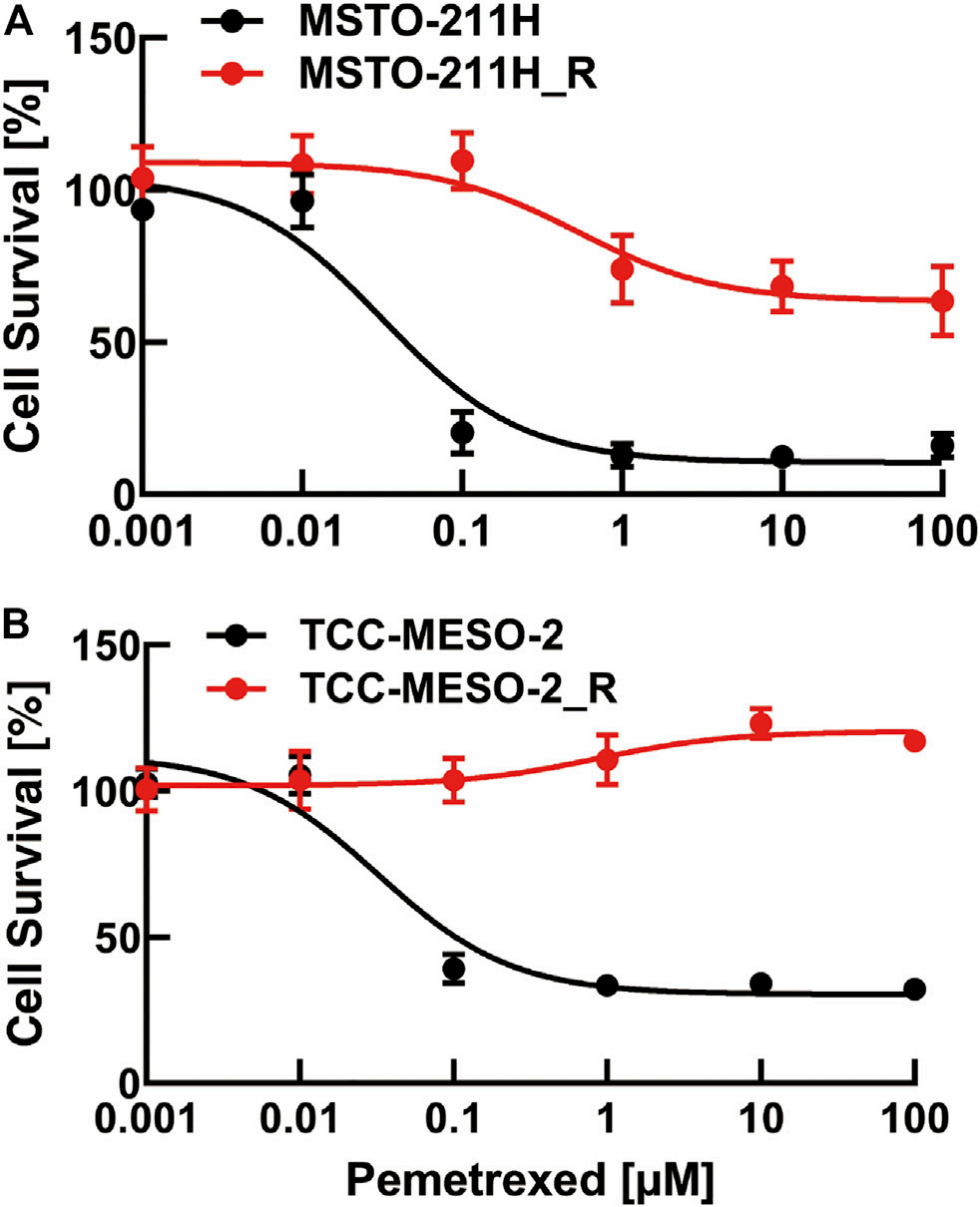
Viability of pemetrexed (PMX)-treated parental and PMX-resistant cells. **(A)** MSTO-211H and MSTO-211H_R and **(B)** TCC-MESO-2 and TCC-MESO-2_R cells were treated with the indicated concentration of PMX for 72 h, and their viability was assessed by WST-8 assay. The percentage (%) viability data are shown as the mean ± standard deviation (SD) (*n* = 6). Error bars indicate the range of SD.

First, we established two resistant cell lines to PMX, based on Buque’s paper (Buque et al., 2013). We measured the number of viable cells after PMX treatment by performing proliferation assays, in order to evaluate the drug sensitivities of the parental and resistant cell lines. We constructed dose–response curves for PMX-treated parental and resistant cells to determine cell growth inhibition (Figure 1). The sensitivity of human MPM cells to PMX was analyzed using a WST-8 cell-counting assay after 72 h of exposure. The IC_50_ value was defined as the dose of PMX required to reduce the viability of MPM cells by 50%. The IC_50_ values for all four MPM cell lines after 72 h PMX treatment were as follows: MSTO-211H: 31.8 nM, MSTO-211H_R: 413.6 nM, TCC-MESO-2: 32.3 nM, and TCC-MESO-2_R: 869.2 nM. The IC_50_ values were 13.0- and 28.0-fold higher in the PMX-resistant MSTO-211H_R and TCC-MESO-2_R cell lines, respectively. In contrast, no significant difference was found between the sensitivities of the parental and PMX-resistant cells to 5-FU, a typical TYMS-targeting drug (Supplementary Figure S1). Therefore, the acquired drug resistance in these cells was specific to PMX. Thus, we established two PMX-resistant cell lines with greater resistance to PMX than the parental cell lines.

### Pemetrexed-Resistant Cells Showed Significantly Higher Thymidylate Synthase Expression

**FIGURE 2:**
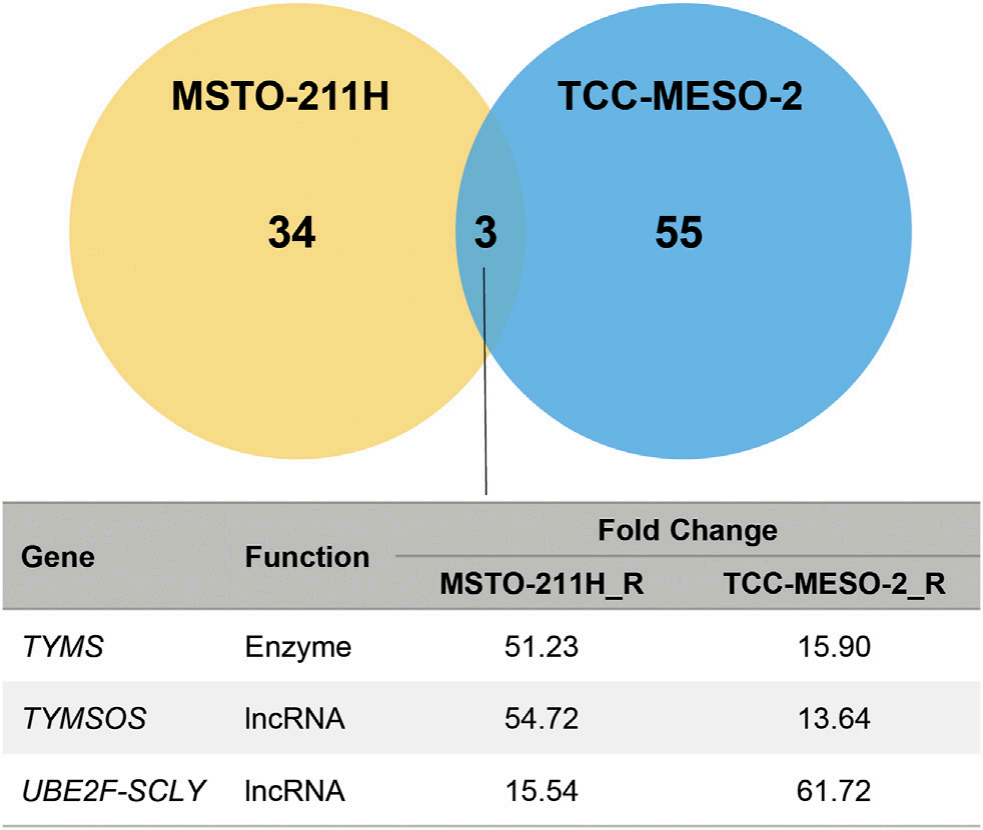
Acquired resistance to pemetrexed significantly increased the expression of *TYMS*. RNA-seq in MPM cell lines identified the differentially expressed genes (DEGs) between parental (MSTO-211H and TCC-MESO-2) and versus resistant (MSTO-211H_R and TCC-MESO-2_R) cell lines, with significant difference in the expression of three genes in both cells (criteria: |Fold Change| > 2; q-value < 0.05). *TYMS*, thymidylate synthase; *TYMSOS*, thymidylate synthase opposite strand; *UBE2F-SCLY*, ubiquitin-conjugating enzyme E2F and selenocysteine lyase; lncRNA, long noncoding RNA.

To identify genes that increase resistance to PMX, MPM cells were comprehensively analyzed using RNA-seq. The raw data were subjected to differential gene-expression analysis, and differentially expressed genes (DEGs) between the established PMX-resistant cells and their respective parental cells were identified (Figure 2). We found that the expression levels of three genes were significantly increased in the resistant cell lines when compared to those in the respective parental cell lines (*TYMS*: MSTO-211H_R: 51.23-fold, TCC-MESO-2_R: 15.90-fold; thymidylate synthase opposite strand (*TYMSOS*): MSTO-211H_R: 54.72-fold, TCC-MESO-2_R: 13.64-fold; ubiquitin-conjugating enzyme E2F and selenocysteine lyase (*UBE2F-SCLY*): MSTO-211H_R: 15.54-fold, TCC-MESO-2_R: 61.72-fold). Interestingly, genes that were previously reported to be associated with drug resistance (*ABCC1–5* and *RFC*) were not differentially expressed (*p* > 0.05).

To validate the RNA-seq results, we evaluated the expression levels of the three DEGs by western blotting and RT-qPCR analyses. We confirmed that the expression level of TYMS was higher in the resistant cell lines than in the respective parental cell lines. However, DHFR expression was only higher in TCC-MESO-2_R cells versus the parental cells, whereas GART expression did not differ between the parental and resistant cell lines (Supplementary Figure S2A).

Similarly, RT-qPCR analysis revealed that *TYMS* mRNA-expression levels were significantly higher in both PMX-resistant cell lines than in the respective parental cell lines (MSTO-211H_R/MSTO-211H: 35.8-fold, TCC-MESO-2_R/TCC-MESO-2: 16.4-fold). However, the *DHFR* and *GART* mRNA-expression levels were not significantly different between the parental and PMX-resistant cell lines (Supplementary Figure S2B).

These findings suggest that increased TYMS mRNA and protein expression was associated with PMX resistance. Therefore, among the three PMX-targeted enzymes identified by RNA-seq, we focused on TYMS in subsequent experiments.

### Thymidylate Synthase Expression Altered Drug Sensitivity

**FIGURE 3:**
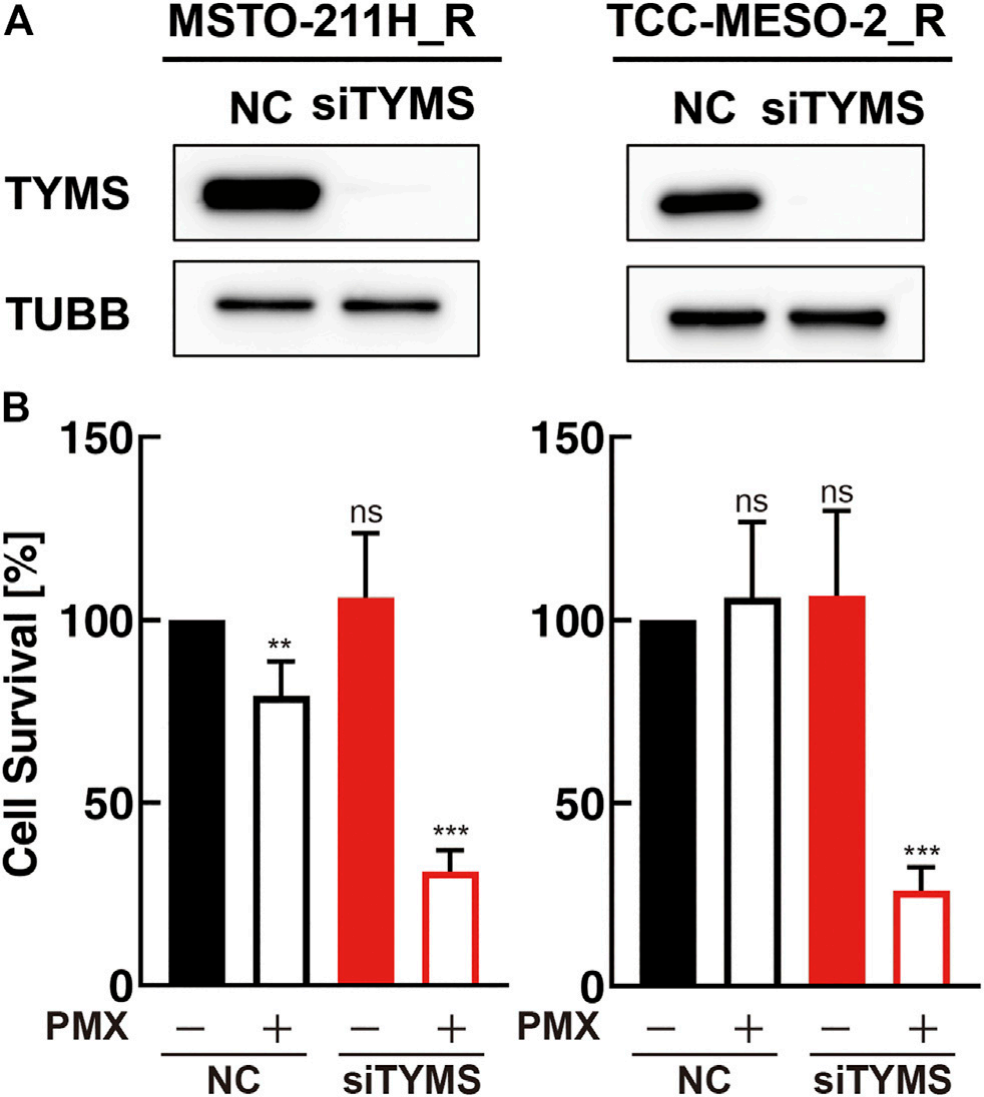
*TYMS* knockdown decreased resistance to pemetrexed (PMX). MSTO-211H_R or TCC-MESO-2_R cells were transfected with either negative control siRNA (NC) or *TYMS* siRNA (siTYMS). **(A)** Protein levels of TYMS or β-tubulin (TUBB) in whole-cell lysates were determined by western blot analysis. TUBB was used as a loading control. **(B)** Viability of transfected cells treated with PBS or PMX (1 μM) for 72 h was determined by WST-8 assay. The % viability data are shown as the mean ± standard deviation (SD) (*n* = 6). Error bars indicate the range of SD. ns; not significant, *p < 0.05, **p < 0.01, ***p < 0.001 vs. control (NC, PMX-) by One-Way ANOVA using for graph pad prism.

As we found that the mRNA- and protein-expression levels of TYMS increased with the acquisition of PMX resistance, we next investigated the function of TYMS by performing knockdown experiments using siRNA transfections (siTYMS and NC) in MSTO-211H_R and TCC-MESO-2_R cells. Western blot analysis confirmed that both resistant cell lines transfected with siTYMS had lower TYMS protein-expression levels than those transfected with NC (Figure 3A). Upon treating the transfected cells with PMX, we found that both resistant cell lines with *TYMS* knockdown showed decreased PMX resistance than the NC siRNA-treated cells (Figure 3B). On the other hand, there were no significant differences between siTYMS-treated cells and NC siRNA-treated cells in terms of cell viability in the absence of PMX.

**FIGURE 4:**
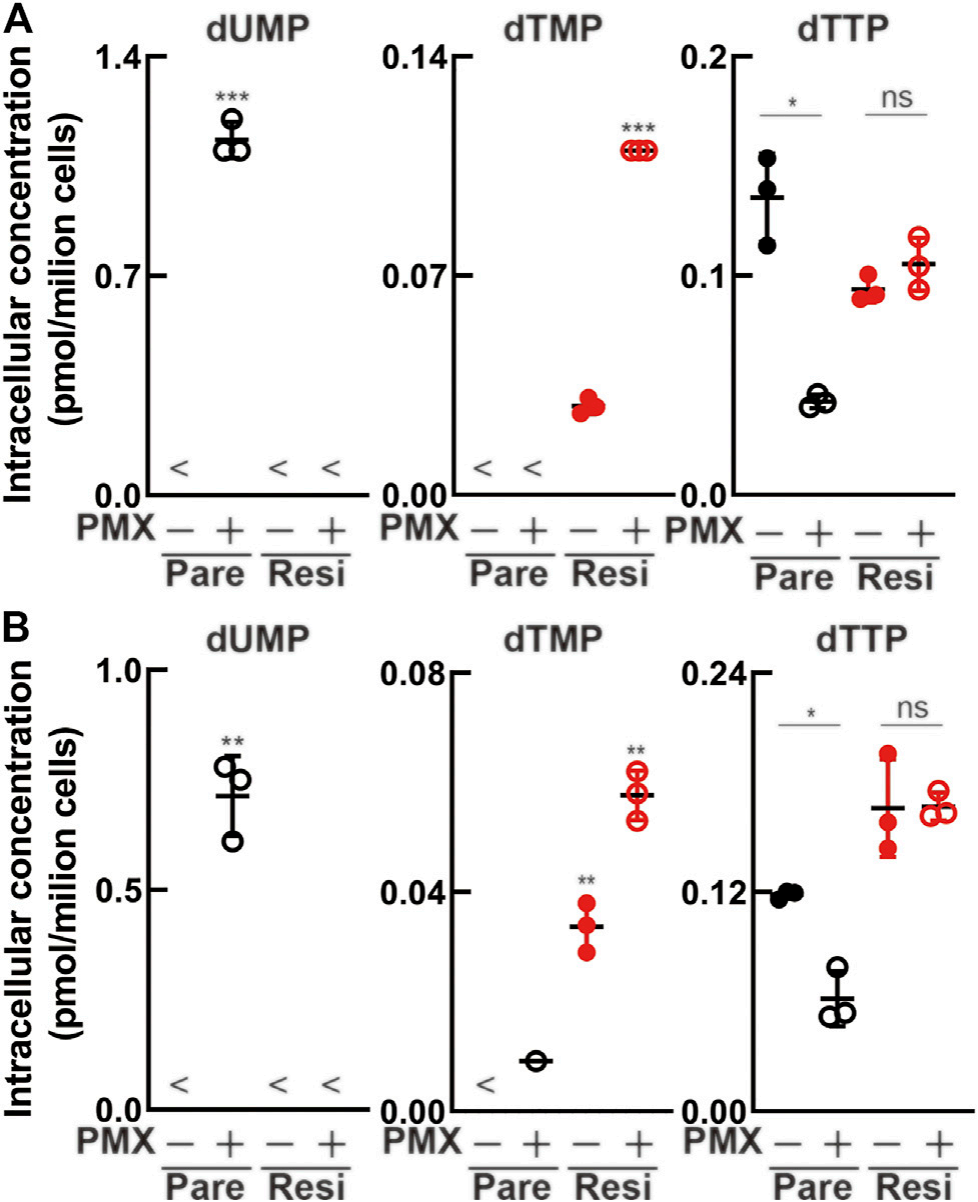
*TYMS* overexpression increased resistance to pemetrexed (PMX). MSTO-211H or TCC-MESO-2 cells were transfected with control vector (Vector) or TYMS-overexpressing vector (TYMS+) derived from retrovirus. **(A)** Protein levels of TYMS or β-tubulin (TUBB) in whole-cell lysates, determined by western blot analysis. TUBB was used as a loading control. **(B)** Viability of transfected cells treated with the indicated concentration of PMX for 72 h, determined by WST-8 assay. The % viability data are shown as the mean ± standard deviation (SD) (n = 6). Error bars indicate the range of SD.

Next, we investigated whether drug resistance was altered by *TYMS* overexpression due to retroviral transduction. Western blot analysis showed that both parental cell lines transduced with a TYMS-overexpression vector had higher TYMS expression than the respective control vector-transduced cells (Figure 4A). We found that TYMS-overexpressing parental cells showed greater resistance to PMX treatment than the respective control vector-transduced cells (Figure 4B). These results showed that both TYMS overexpression and silencing affected PMX resistance in parental cell lines.

### Altered Intracellular Metabolite Concentration due to Acquired Pemetrexed Resistance

**FIGURE 5:**
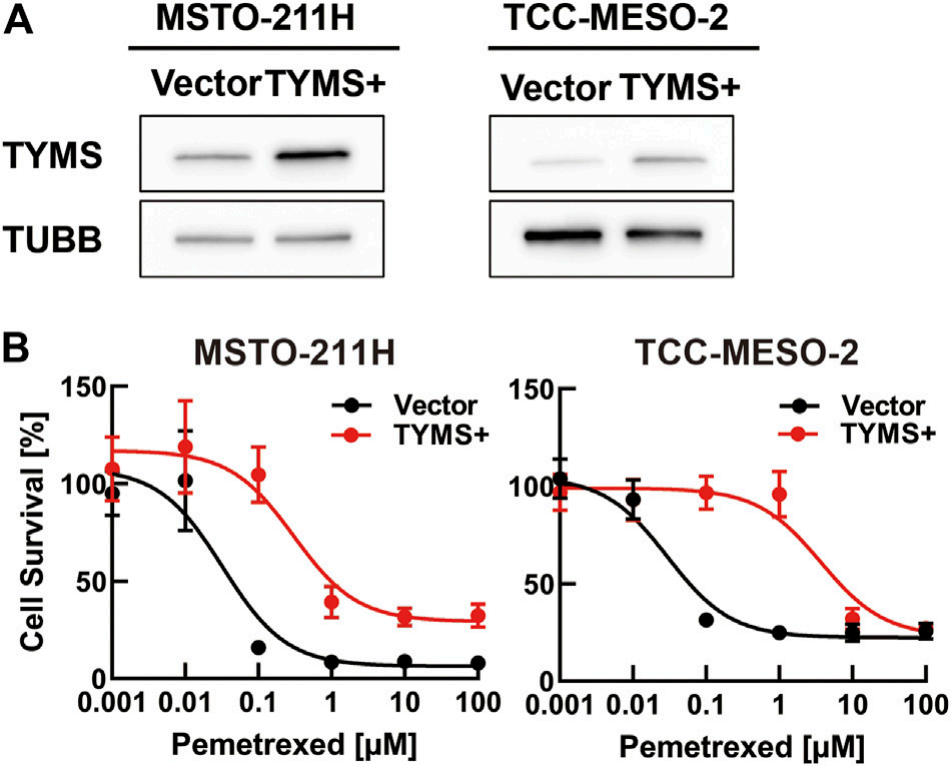
Intracellular metabolite levels in parental and PMX-resistant cells treated with PMX. Intracellular concentration (pmol/10^6^ cells) of key metabolites involved in the nucleotide biosynthesis in **(A)** MSTO-211H vs. MSTO-211H_R and **(B)** TCC-MESO-2 vs. TCC-MESO-2_R cells after treatment with PBS or PMX (1 μM) for 6 h. Error bars indicate the range of SD. *p < 0.05, **p < 0.01, ***p < 0.001 vs. control (PBS) by Welch’s t-test.

We performed metabolomics analysis to evaluate whether drug resistance acquisition could suppress the antiproliferative effect of PMX. Following treatment with PBS or PMX for 6 h, intracellular metabolites in the four MPM cell lines (parental and resistant) were extracted with methanol and analyzed using CE-TOFMS (Figure 5, Supplementary Tables S1, S2). There was dUMP accumulation after PMX treatment in the two parental cell lines. However, dUMP was not detected in either of the PMX-resistant cell lines. dTMP was detected in both resistant cell lines treated with PBS (control), and the dTMP level increased after PMX treatment. We found that dTTP was reduced in both parental cell lines after PMX treatment. However, PMX treatment did not alter the dTTP levels in the resistant cell lines. Based on these findings, we believe that PMX-resistant cells may escape PMX-induced TYMS inhibition due to elevated TYMS expression, thus generating a dTMP pool that helps them escape dTTP reduction after PMX treatment.

### Thymidylate Synthase Expression Increased due to Histone Acetylation

Because TYMS expression is reportedly regulated by FOXM1, c-MYC, and E2F1, we detected these proteins in whole-cell lysates of MPM cells by western blotting (Supplementary Figure S3). The expression levels of these proteins did not change following the acquisition of drug resistance or *TYMS* knockdown. Therefore, we hypothesized that the increased TYMS expression was caused by loosening of the histone structure and histone modification on the 5′-UTR of *TYMS*.

**FIGURE 6:**
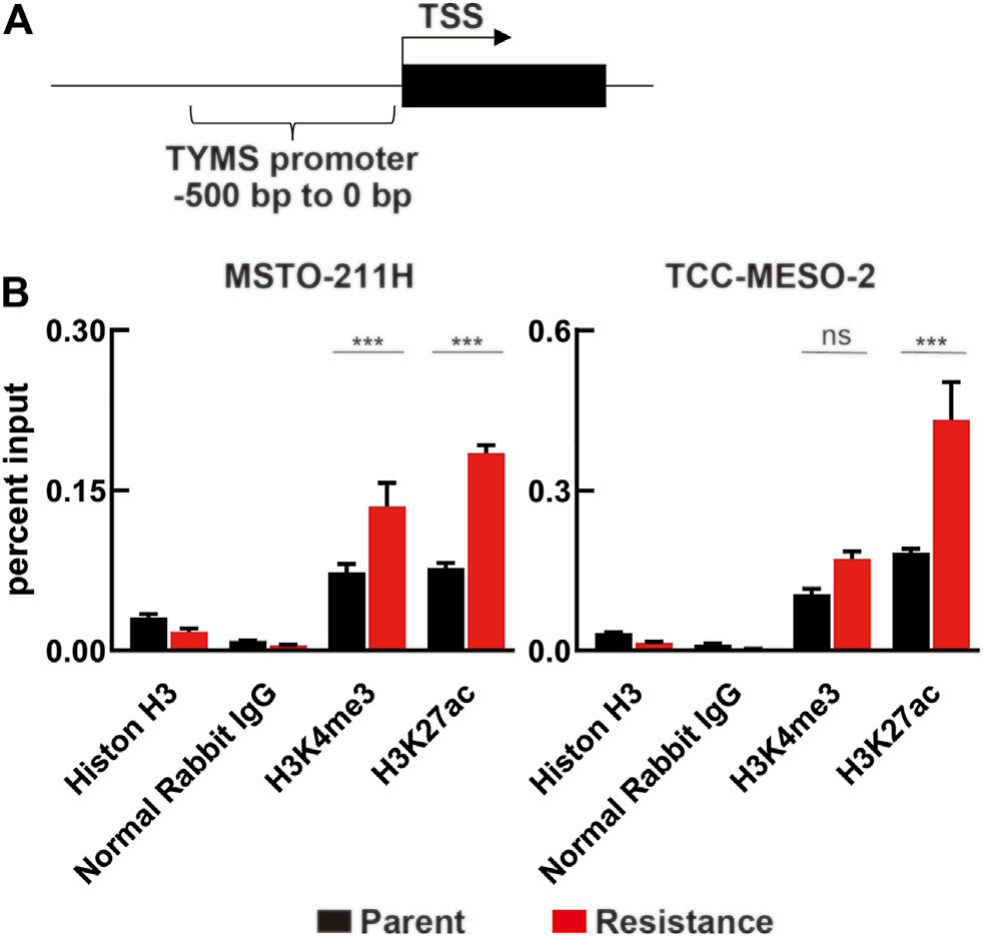
Changes in histone modification due to PMX-resistance in the *TYMS* promoter. **(A)** Schematic of the region 0–500 bp upstream of the *TYMS* transcription start site (*TYMS* TSS). **(B)** Chromatin in MPM cells were immunoprecipitated using antibodies against Histone H3, Normal Rabbit IgG, H3K4me3, and H3K27ac, and the TYMS TSS (−500 to 0 bp) mRNA expression was evaluated by real-time RT-PCR and normalized to input DNA. Error bars indicate the range of SD. ns, not significant, *p < 0.05, **p < 0.01, ***p < 0.001 vs. parent cell by Two-way ANOVA using for graph pad prism.

Based on this possibility, we further hypothesized that H3K4me3 and H3K27ac, which have been reported to be involved with the transcription start site (TSS) of actively transcribed genes near the promoter region (Kimura, 2013), participate in loosening the chromatin structure upstream of the *TYMS* TSS (Figure 6A, Supplementary Figure S4). To test this hypothesis, we performed ChIP experiments and analyzed the samples using ChIP-qPCR. We found a significant difference in H3K27ac levels between parental and resistant cell lines (Figure 6B). However, H3K4me3 levels were only significantly different in MSTO-211H cells. On the other hand, we evaluated whether short-term treatment with PMX altered histone modification in MPM cells, but we found no change (Supplementary Figure 5). Therefore, we propose that long-term exposure of MPM cells to PMX causes H3K27 acethylation near the *TYMS* TSS, resulting in increased *TYMS* expression.

## Discussion

Antifolates represent a feasible chemotherapeutic option for treating MPM. Antifolates, such as PMX, primarily target TYMS and are still used as chemotherapeutic agents for many cancers. Previously, increased TYMS expression has been reported as one of the major factors leading to PMX resistance (Sigmond et al., 2003; Takezawa et al., 2011; Zhang et al., 2011). This study confirmed that acquired PMX resistance in MPM cells was caused by increased TYMS expression. Additionally, we showed that acquired drug resistance may lead to the cancellation of dUMP accumulation and the formation of dTMP pools due to increased TYMS expression in MPM cells. We found that this increase in TYMS expression was caused by histone H3K27 acetylation in the 5′-UTR near the *TYMS* TSS. Consequently, we determined that the mechanism underlying the increase in TYMS expression involved H3K27 acetylation, which loosens the histone structure of TYMS and promotes its transcription.

A previous study showed that cells treated with PMX had depleted intracellular dTTP levels (Chen et al., 1998). Our results showed that dTTP decreased in the parental MPM cell lines after PMX treatment, consistent with previous results. Furthermore, we found that dUMP accumulated in the parental MPM cell lines after PMX treatment. In contrast, dTTP concentration was maintained in resistant cell lines, even when TYMS expression was increased. These findings suggest that intracellular dTTP concentration might be more tightly controlled in resistant cell lines than in parental cell lines and may be attributable to the acquisition of drug resistance. We believe that this result highlights the importance of metabolomics analysis.

Data from a previous study showed that TYMS expression might not necessarily be associated with drug resistance (Kitazono-Saitoh et al., 2012). On the other hand, a previous clinical study reported that TYMS expression was not a predictable marker of PMX efficacy as therapy (Lustgarten et al., 2013). We have previously reported that NCI-H2452 cells have PMX resistance and expressed significantly lower TYMS levels than the susceptible cell lines used in the present study (Sato et al., 2018). Therefore, we consider that the mechanisms of congenital and acquired drug resistance may differ in MPM cells. That is, we hypothesize that congenital PMX resistance is independent of TYMS expression. We focus on our results that intracellular dTMP levels detectd in PMX-resistant cells. Because, there are known for dTMP synthesize also involved tymidine kinase. We expect that biopsy samples will be available for evaluation dTMP levels. Metabolomics observations may reveal a mechanism of drug resistance in TYMS-independent cells.

As shown in Figure 4B, MPM cells with *TYMS* overexpression due to retroviral transduction had lower PMX resistance compared to the results in Figure 1. This phenotype was especially shown when TCC-MESO-2 cells with TYMS overexpression were treated with high concentrations of PMX. We speculated that these results were because MPM cells with TYMS overexpression were lower than established resistant cells (Figure 4B, Supplementary Figure S2A). Moreover, we surmised that other genes may be in coordination with TYMS.

In this study, we showed that the exposure of MPM cells to PMX lowered intracellular dTTP levels and suppressed cell proliferation. Moreover, from the results of prolonged exposure of MPM cells to PMX, we showed that TYMS expression increased in MPM cells due to the induction of H3K27 acetylation to solve this problem. However, the mechanism of H3K27 acetylation induction and the involvement of transcription factors in the TYMS promoter region were not determined. It has previously been reported that histone deacetylase 1,3,7 (HDAC) promotes H3K27 acetylation in the TSS region in breast cancer cells (Caslini et al., 2019). Additionally, it has been reported that H3K27 acetylation suppresses H3K27 methylation in lymphoma cells (Knutson et al., 2012) and solid tumor cells (Huang et al., 2018). We could not find via RNA-seq in this study that the altered expression of genes associated with histone modification and transcription by the acquisition of PMX resistance. Single-cell analyses, such as single-cell RNA-seq and single-cell ATAC-sequencing (Kashima et al., 2021), are necessary to make this finding. In addition, it will be necessary to investigate increased TYMS expression-associated histone modification in more detail using ChIP-sequencing, and research related to DNA methylation (Charlet et al., 2016; Onuchic et al., 2018) may also be necessary.

In summary, our results link the acquisition of antifolate resistance to increased TYMS expression and indicate that the intracellular levels of dTMP may be potential biomarkers for PMX treatment. Further studies on epigenetic modifications of the *TYMS* gene elucidating its metabolic regulation and related mechanisms will be useful, not only for developing new therapies for MPM but also for other cancers.

## Data Availability

The datasets presented in this study can be found in online repositories. The names of the repository/repositories and accession number(s) can be found below: DNA Data Bank of Japan (DDBJ) Accessions DRA012402 - DRA012406.

## Glossary

5′-UTR: 5′-untranslated region
ABCC: ATP-binding cassette subfamily C
cDNA: complementary DNA
CE-MS/MS: capillary electrophoresis-tandem mass spectroscopy
CE-TOFMS: capillary electrophoresis-time-of-flight mass spectrometry
ChIP: chromatin immunoprecipitation
CST: Cell Signaling Technology
Ct: cycle threshold
DHFR: dihydrofolate reductase
DMEM: Dulbecco’s modified Eagle’s medium
dTMP: deoxythymidine monophosphate
dTTP: deoxythymidine triphosphate
dUMP: deoxyuridine monophosphate
E2F1: E2F transcription factor 1
FBS: fetal bovine serum
FOXM1: forkhead box M1
GAPDH: glyceraldehyde 3-phosphate dehydrogenase
GART: glycinamide ribonucleotide transformylase
lncRNA: long noncoding RNA
MPM: malignant pleural mesothelioma
MYC: MYC proto-oncogene, bHLH transcription factor
NC: negative control
PBS: phosphate-buffered saline
RFC: reduced folate carrier
RNA-seq: RNA sequencing
RT-qPCR: reverse transcriptase-polymerase chain reaction
siRNA: small-interfering RNA
siTYMS: TYMS siRNA
TSS: transcription start site
TYMS: thymidylate synthase
TYMSOS: thymidylate synthase opposite strand
UBE2F-SCLY: ubiquitin-conjugating enzyme E2F and selenocysteine lyase
WST-8: water-soluble tetrazolium salt-8
