# Supplementary figures and images for: Upregulation of Thymidylate Synthase Induces Pemetrexed Resistance in Malignant Pleural Mesothelioma
Source: Front Pharmacol. 2021 Sep 27;12:718675. doi: 10.3389/fphar.2021.718675 (PMC8504579; doi:10.3389/fphar.2021.718675)

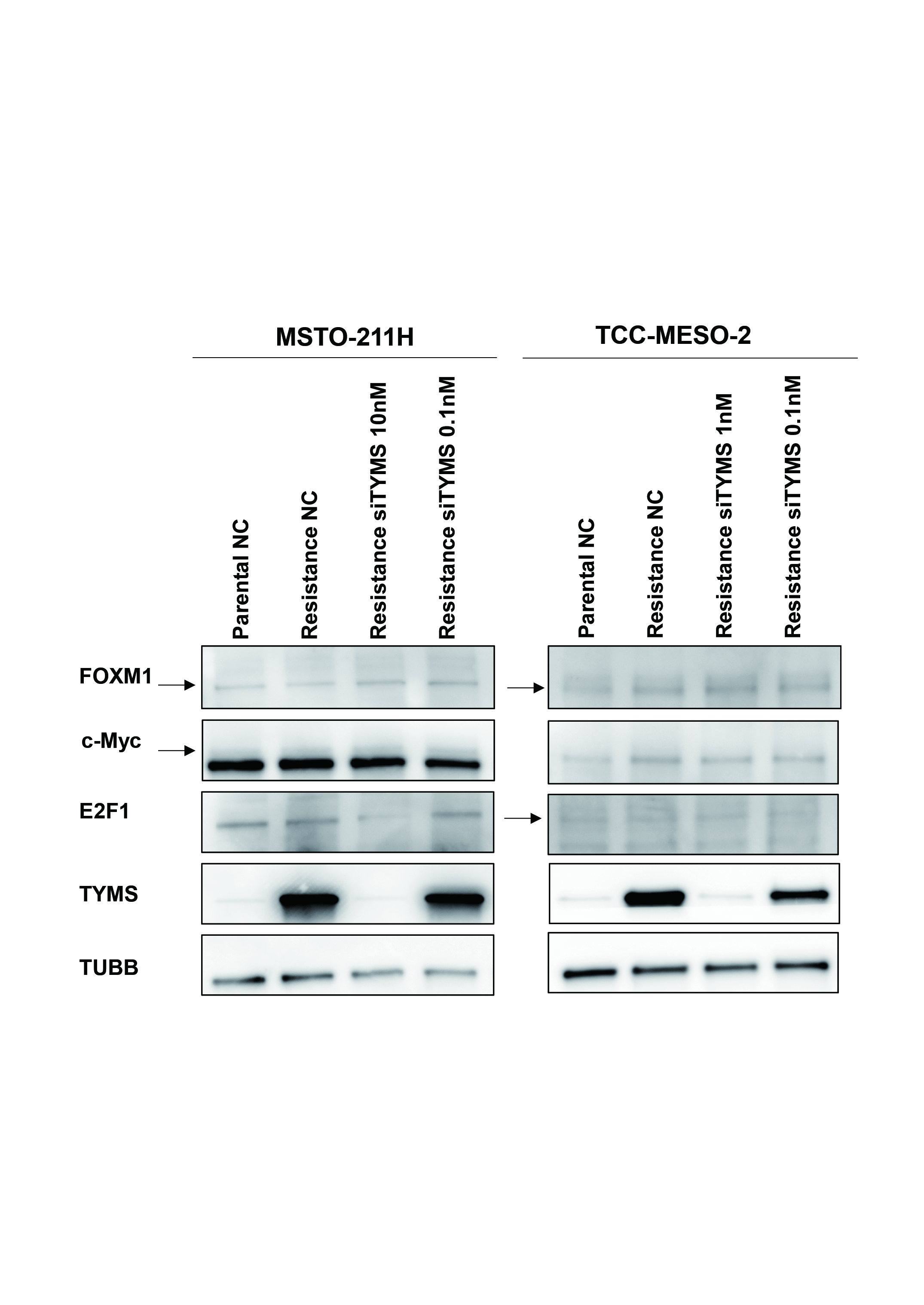

Supplement: Supplementary file 2 [file Image3.TIF]

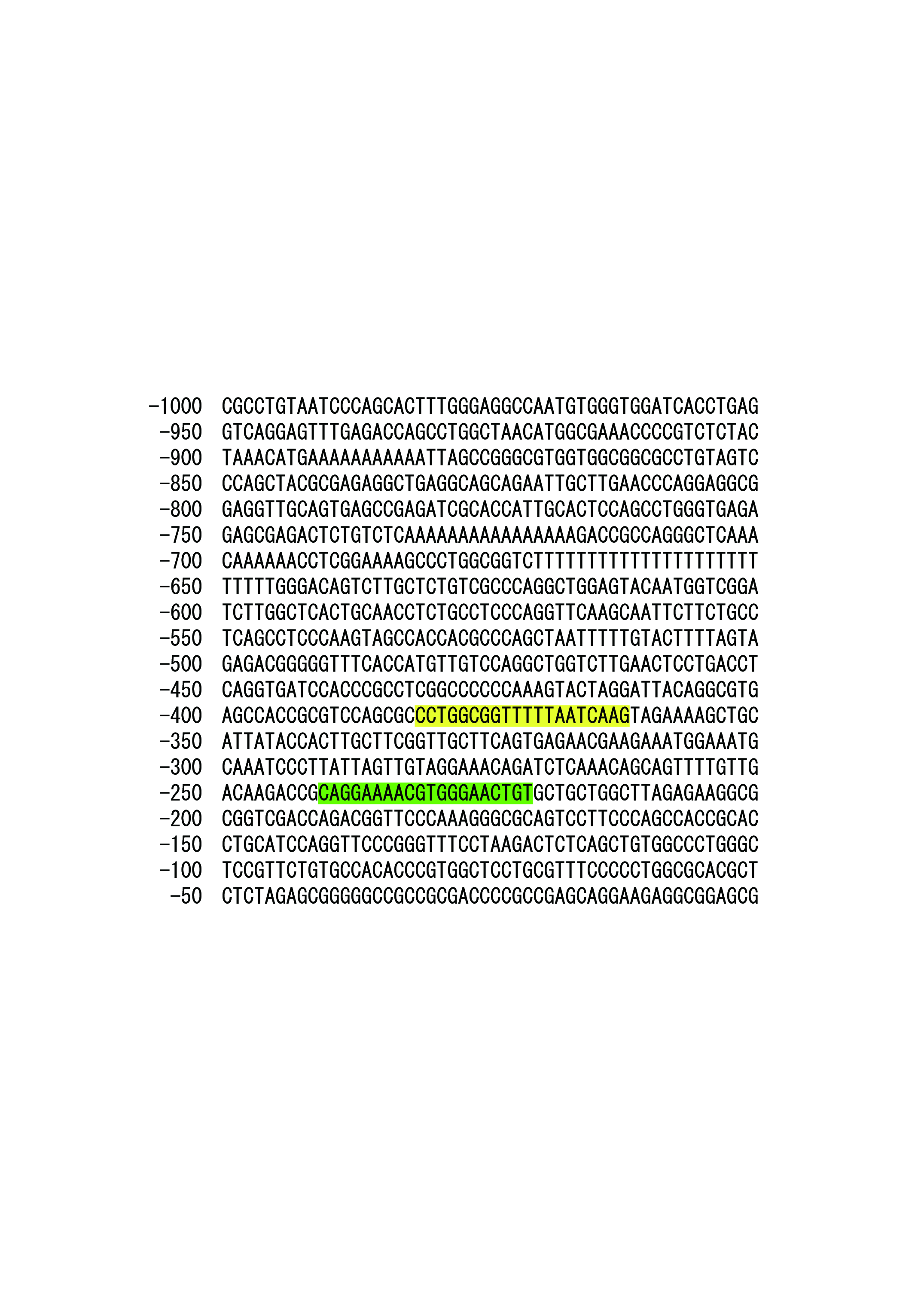

Supplement: Supplementary file 3 [file Image4.TIF]

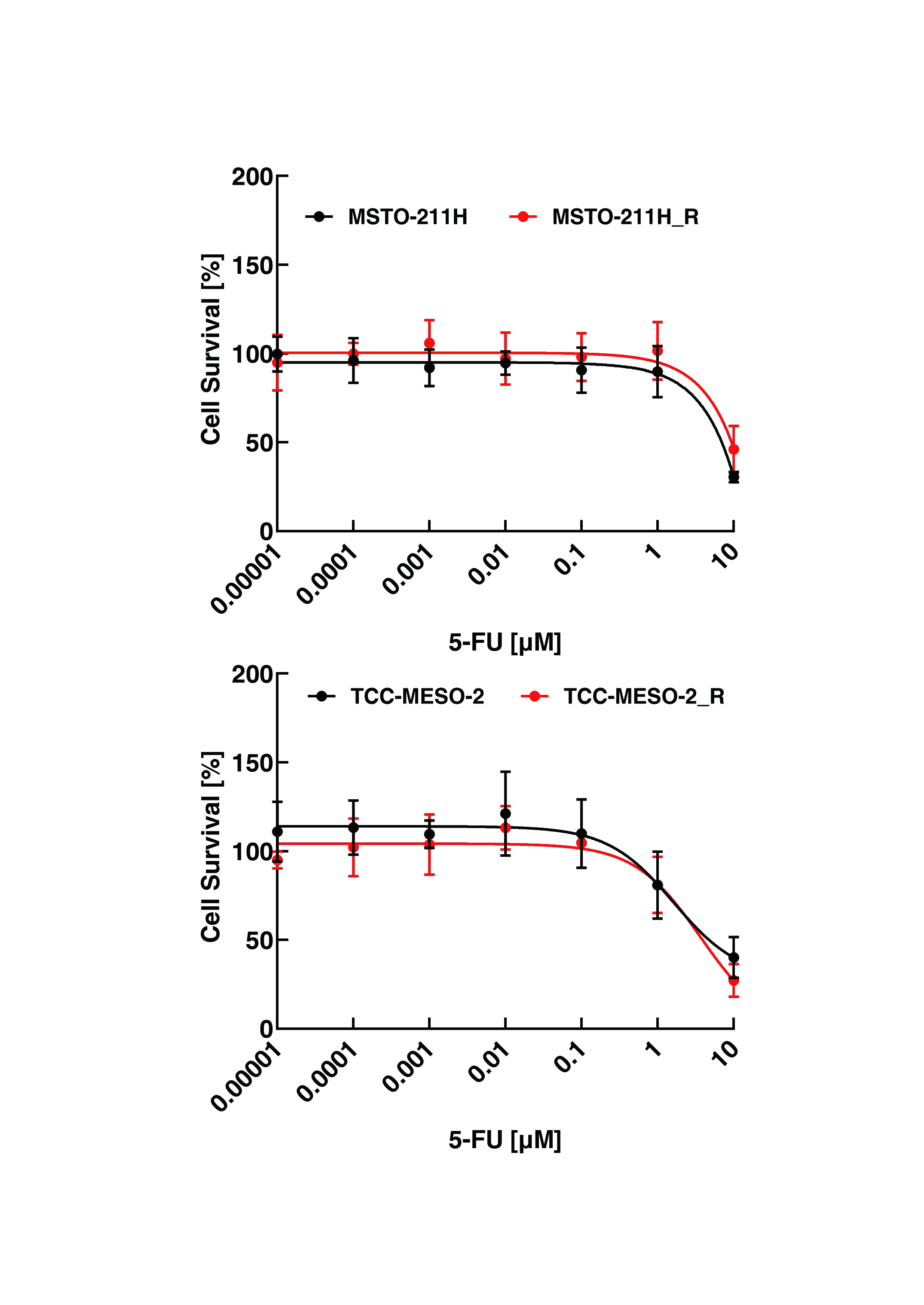

Supplement: Supplementary file 5 [file Image1.TIF]

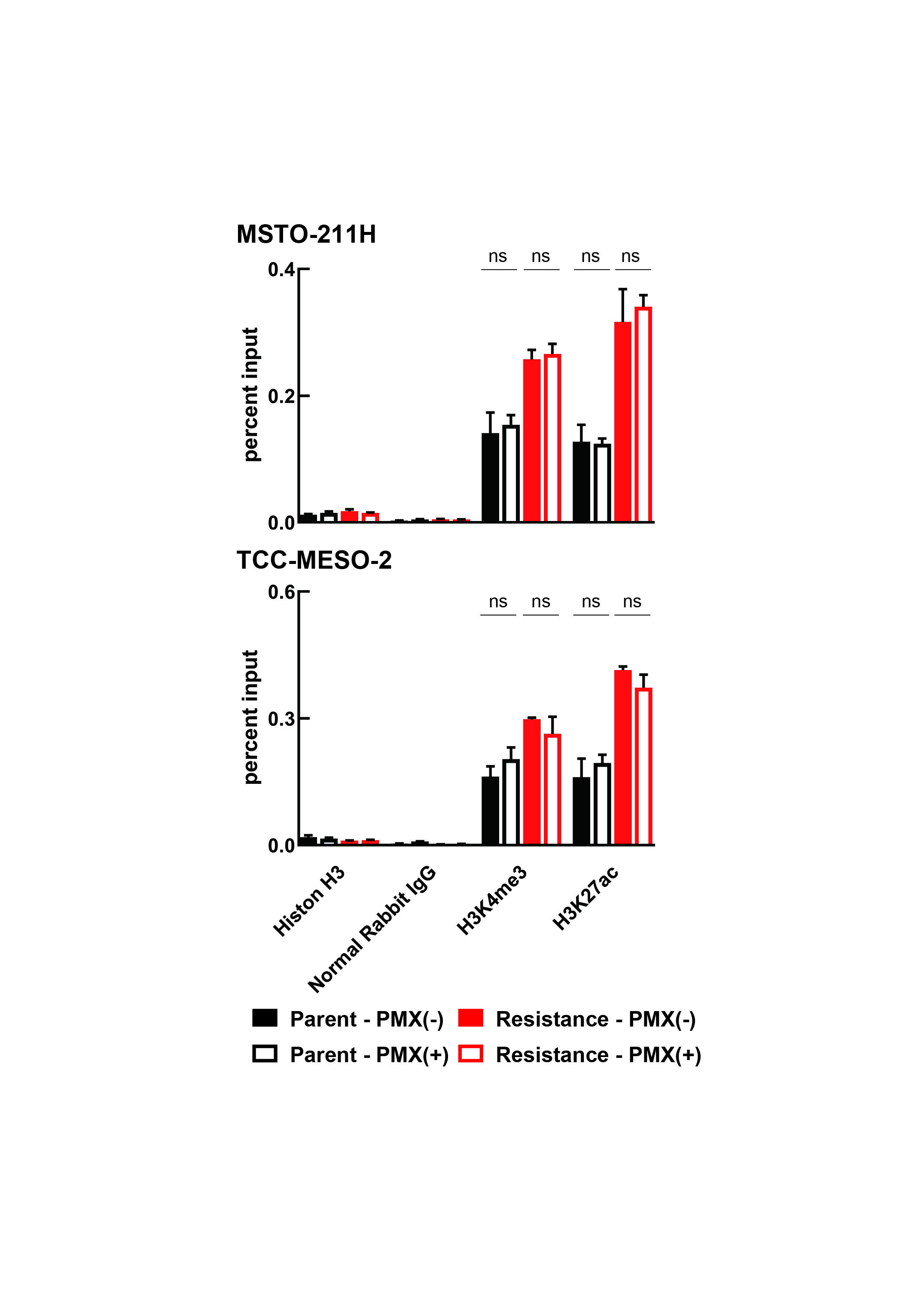

Supplement: Supplementary file 7 [file Image5.TIF]
